# A cross-sectional assessment of knowledge, awareness of risk factors, and perceptions of thyroid disease (TD) among adults living in Saudi Arabia – A community based study

**DOI:** 10.3389/fpubh.2022.1041745

**Published:** 2022-11-23

**Authors:** Riyadh A. Alhazmi, Abdullah M. Alobaid, Saqer Mohammed Althunayyan, Wajid Syed, Mahmood Basil A. Al-Rawi

**Affiliations:** ^1^Emergency Medical Service Department, Prince Sultan bin Abdulaziz College for Emergency Medical Services, King Saud University, Riyadh, Saudi Arabia; ^2^Department of Trauma and Accident, Prince Sultan bin Abdulaziz College for Emergency Medical Services, King Saud University, Riyadh, Saudi Arabia; ^3^Department of Clinical Pharmacy, College of Pharmacy, King Saud University, Riyadh, Saudi Arabia; ^4^Department of Optometry, College of Applied Medical Sciences, King Saud University, Riyadh, Saudi Arabia

**Keywords:** thyroid disease, neck pain, awareness, risk factors, treatment, Saudi Arabia

## Abstract

**Background:**

The incidence of thyroid diseases has tripled in the last three decades, and the prevalence is rising rapidly irrespective of gender and genetics. This study aimed to assess the Knowledge, awareness of risk factors, and perceptions of thyroid disease among the Saudi Community in Saudi Arabia.

**Methods:**

A cross-sectional, online web-based, survey type study was conducted between November 2021 to January 2022 among residents living in Saudi Arabia. Individuals aged ≥ 18 years who expressed a willingness to complete the survey were included. Descriptive and bivariate analyses were carried out to determine the factors associated with knowledge of thyroid using SPSS version 26.0 software (SPSS Inc., Chicago, IL, U.S.).

**Results:**

Among the participants, the majority of them were females than males (77.5 vs. 22.5%). A total of 78.2% (*n* = 566) of them were aware of the thyroid. Nearly 44% (*n* = 312) of respondents are aware that a lump in the neck or swelling is a sign of thyroid disease, followed by pain in the neck 24.6% (*n* = 178), and difficulty in swallowing 23.8% (*n* = 172). The mean knowledge score of the thyroid was 4.1 (SD = 3.09), while the score of the mean perception was 33.02 (SD = 6.41). The mean knowledge scores were significantly associated with having previous knowledge of thyroid disease (*t* = 5.08; *p* = 0.0001). The gender of the participant and the presence of chronic diseases were found to have no impact on the knowledge score of the thyroid disease (*t* = −1.18; *p* = 0.235; *t* = 1.005; *p* = 0.315). Additionally, the perceptions score was not significantly associated with the demographics of the participants (*p* = 0.05).

**Conclusion:**

In this study, Saudi adults reported varying levels of knowledge and perceptions of thyroid disease. Having previous knowledge of the thyroid was significantly associated with the knowledge score. It is necessary to educate people about this rising disease.

## Introduction

The thyroid gland is a butterfly-shaped gland situated in the neck that regulates many metabolic processes and physiological functions in the body (1, 2). In recent years, abnormal thyroid function has been a major problem in clinical practice, raising health concerns among patients (3). Thyroid illness is caused by a deficiency in iodine or by autoimmune diseases (4, 5). Other studies have shown that thyroid disease is caused by inflammation or particular medical operations such as radiation or thyroid surgery or by a hereditary factor (1, 2). Thyroid diseases are caused by excessive or insufficient thyroid hormone secretion, as well as thyroid gland hypertrophy. It is estimated that one-third of the world's population suffers from iodine deficiency, with 1.6 billion people at risk of developing thyroid disorders (6). Furthermore, previous studies discovered that physical stress is a possible cause of thyroid gland dysfunction. The most prevalent indications and symptoms of an underactive thyroid gland were fatigue, dry skin, weight gain or loss, changes in bowel movement and menstruation cycle, hair loss and abnormal metabolism and growth, and myalgia (7).

Internationally, the prevalence of thyroid is on the rise, and incidence were more in females than males. The prevalence of thyroid among middle-aged women was 7% (8), the most common form of the disease is the thyroid nodule, accounting for 19–68% of the general population, more commonly found in women (9). According to recent estimates in Saudi Arabia, the prevalence of thyroid dysfunction was 49.76%, and subclinical hypothyroidism was the most prevalent type at 39.2% among Saudis, while primary hypothyroidism was reported at 5.3% (5). The clinical signs of thyroid are primarily determined by the type of thyroid (hypothyroidism or hyperthyroidism or thyroid nodules) and can alter multiple physiological functions in the body including metabolism. Thyroid diseases are also often neglected or mistaken for other medical conditions because most of the symptoms are non-specific (10).

There were previous studies that estimated knowledge, attitudes, and practice toward influenza and diabetes nationally (11, 12), while international studies assessed the knowledge of thyroid cancer (13), and only a few studies evaluated the knowledge, practice, and prevalence of thyroid diseases in Saudi Arabia (1, 5, 6, 14, 15). Although the majority of earlier studies were carried out in other parts of Saudi Arabia, our study stands out for its original design and sample, complete attention to the thyroid, clinical information related to it, manifestations, symptoms, and other factors. As a result, it raises public awareness of the hidden thyroid. Since thyroid disorders are one of the most under-diagnosed and neglected medical conditions, and the lack of general knowledge among patients may be of considerable concern, posing adequate knowledge about the disease is likely an important element in curtailing the prevalence (15). There are only limited studies that evaluated the awareness and knowledge on preventive practices toward thyroid disease in Saudi Arabia from community perspectives. Previously published studies in Saudi Arabia reported a lack of knowledge despite the increased prevalence of disease, which would greatly influence the morbidity and mortality rates. Therefore, this study aimed to assess the knowledge, awareness of risk factors, and perceptions of thyroid disease among the Saudi community in Saudi Arabia.

## Methods

### Study design, settings, and population

This was a cross-sectional study conducted using an online self-reporting questionnaire among the Saudi Community in Saudi Arabia over 3 months, from November 2021 to January 2022. Saudi adults aged between 18 and 50 years and above, currently living in Saudi Arabia, and willing to provide informed consent were included in the study. We excluded Non-Arabic speakers or those who were outside of the country during the study period. Before data collection, the study protocol was reviewed and approved by the Institutional Review Board (E-21-6371), College of medicine, king Saud University, Riyadh Saudi Arabia.

### Study questionnaire, data collection, and source

The questionnaires were prepared after an extensive literature review using previously published similar studies (1, 5, 13–15). The questionnaire used in this study is divided into four categories. Category one includes 7-items that assess demographic characteristics and certain clinical information of participants such as disease history. The second category contains questions about the knowledge of thyroid disease (4 items), assessed using multiple-choice questions. The third category contains questions about the knowledge of warning signs of the thyroid with a total of 11 items, measured on a three-point scale (Yes/No/I do not know). The fourth category contains questions about the awareness and perception regarding a person's chance of developing thyroid (10 items). The responses of the last section were recorded on a 5-point Likert scale ranging from strongly agree to strongly disagree. A score of 5 was given to strongly disagree, 4 to disagree, 3 to neutral, 2 to agree, and 1 to strongly agree. The knowledge scores were calculated by assigning each correct answer a score of 1 and a wrong answer a score of 0, then all the knowledge items were computed to the obtained overall knowledge. The total knowledge was further divided into good, who scored >50% in the knowledge, while poor considered as individuals who scored < 50% in the knowledge items.

We decided to use a non-probability sampling strategy to gather the data from the intended audience. The final study questionnaire was created using Google forms and distributed *via* social media applications using the “snowball” technique, in which each person who signs up for the survey refers to several other people. Participants were given the assurance that their information would only be used for research purposes and would be kept confidential throughout the study. To assure greater reliability and prevent lower response rates, we targeted at least 1,000 people for the data collection process. A statement outlining the importance of participating in the study was made at the beginning. Filling out the survey provided as both an indication of agreement and verbal informed consent. The data was checked to see if it met the inclusion criteria after being obtained. The study did not include any responses that were missing or incomplete.

The designed questionnaire was validated in two steps. First, the initial draft is evaluated by a research expert in the related field, to check the accuracy of the content and flow of the questionnaire. Second, a pilot study was conducted among a randomly selected sample of (*n* = 20) individuals to give their opinions. Reliability was determined using Cronbach's alpha, which was found to be 0.85. The data from the pilot study was not included in the final analysis. The final questionnaire was then distributed using online survey tools.

### Statistical analysis

The data were recorded and analyzed using the Statistical Package for the Social Sciences (SPSS) Version 26 for Windows (SPSS Inc., Chicago, Illinois). Frequencies were reported in numbers and percentages (for categorical variables) and as means and standard deviation (for continuous variables). The association between demographics and knowledge and perception scores of the thyroid were measured using parametric tests. The association between the mean scores and demographics with two groups was assessed using the student's *t*-test, while demographics with more than two groups were assessed by ANOVA. All statistical tests were performed at a significance level of α = 0.05 and a 95 % confidence interval (CI).

## Results

### Demographics and clinical characters of the participants

The study included a total of 724 Saudi adults who responded to the online survey, yielding a response rate of 72.4%. There were 561 (77.5%) women, and 68% (*n* = 492) of them were aged between 18 and 22. About 29% of the 210 participants were housewives, in contrast to 27.6% of the employed participants (*n* = 200). About 40% (*n* = 283) of them claimed to have a decent family income, while 30% (*n* = 221) had an excellent monthly income. The majority of the 647 (89.4%) participants in the study had no history of chronic disease and 78.2% (*n* = 566) were aware of thyroid cancer. Of the total number of participants, 345 (47.7%) belonged to the central region of Saudi Arabia, while 190 (26.2%) were from the west of Saudi Arabia. Detailed information on the demographic characteristics and clinical profiles of the participants is summarized in Table 1.

**Table 1:** Socio-demographic characteristics and clinical profile of the study sample.

**Demographics**	**Frequency (*n*)**	**Percentage (%)**
**Gender**		
Male Female	163 561	22.5 77.5
**Age (full years)**		
18–22 23–25 26–30 31–44 45–50	492 83 48 70 31	68.0 11.5 6.6 9.7 4.3
**Schooling**		
Primary school/ lower Secondary school University degree	264 148 312	36.5 20.4 43.1
**Professional class**		
Working Self-employed/business Housewife Students Looking for employment	200 157 210 34 123	27.6 21.7 29.0 4.7 17.0
**Family income**		
Excellent Good Average Poor	221 283 191 29	30.5 39.1 26.4 4.0
**Presence of chronic disease?**		
Yes No	77 647	10.6 89.4
**Nationality**		
Saudi Non-Saudi	692 32	95.6 4.4
**Did you hear about thyroid disease?**		
Yes No	566 158	78.2 21.8

### Knowledge about early signs of thyroid cancer

**Figure 1:**
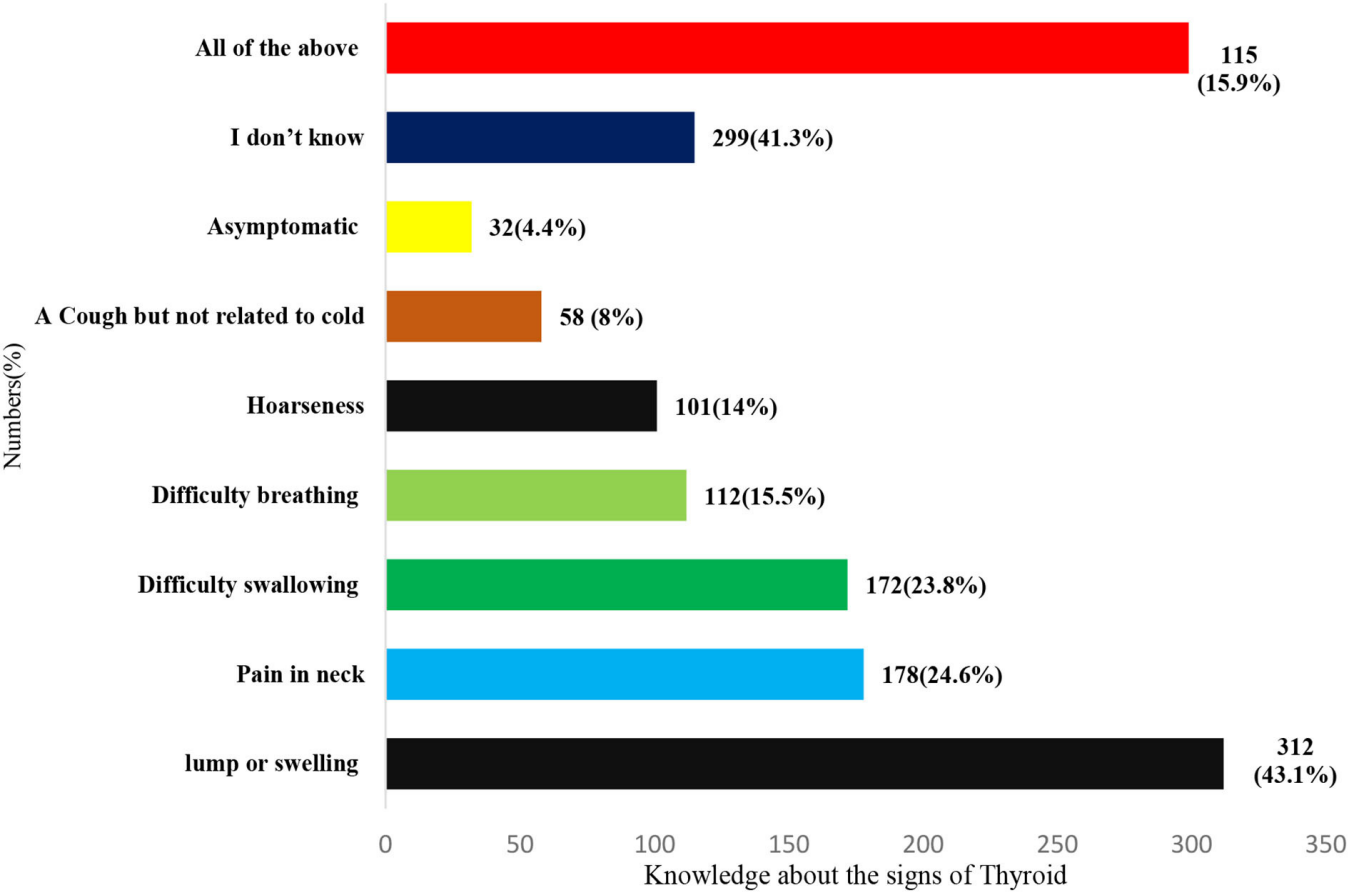
Knowledge about early signs of thyroid cancer.

Figure 1 demonstrates knowledge about the early signs of thyroid cancer. Findings show that roughly 43.1% (*n* = 312) of respondents knew that a lump or swelling in the neck is an indication of thyroid disease, followed by neck pain at a rate of 24.6% (*n* = 178), and difficulty swallowing at a rate of 23.8% (*n* = 172). However, 42% (*n* = 299) of people are unaware of the symptoms and indicators of thyroid disease (Figure 1).

### Participant's responses toward knowledge of thyroid cancer

Table 2 describes the participant's responses regarding the knowledge of thyroid cancer. The majority of participants (39.4%) reported that they had no idea which gender was most affected by the thyroid, while 33.6% reported being female. Only 41.2% (*n* = 291) of the participants in this study gave a favorable response, claiming that thyroid function affects the menstrual cycle, as opposed to 54.1% (*n* = 329) who answered that thyroid function does not affect the menstrual cycle. Over two-thirds of the participants were unaware that thyroid hormone is synthesized using iodine. Knowledge regarding diagnostic methods for screening of thyroid cancer was as follows: 33.4% (*n* = 242) identified neck palpation and thyroid ultrasonography, followed by biopsy by 33.3% (*n* = 241), and hormonal testing by 19.2%(139). When asked about the desirable candidate for the thyroid, most of the participants about 28% (203) reported family history, while high radiation exposure by 11.4% (*n* = 74) (Table 2).

**Table 2:** Participant's responses toward knowledge of thyroid cancer.

**Variables**	**Frequency (*n*)**	**Percentage (%)**
**Most common diagnostic methods for screening for thyroid cancer**		
Neck palpation and thyroid ultrasonography Blood smear Biopsy Hormonal level testing Don't know	242 114 241 139 286	33.4 15.7 33.3 19.2 39.5
**Desirable candidate for a thyroid cancer screen**		
Old age High radiation exposure Family history All of the above I don't know	71 74 203 300 234	9.8 11.4 28 41.4 32.3
**Gender most affected by thyroid dysfunction**		
Male Female Both I don't know	45 243 151 285	6.2 33.6 20.9 39.4

### Frequency of correct answers regarding the Knowledge of warning signs for thyroid

**Figure 2:**
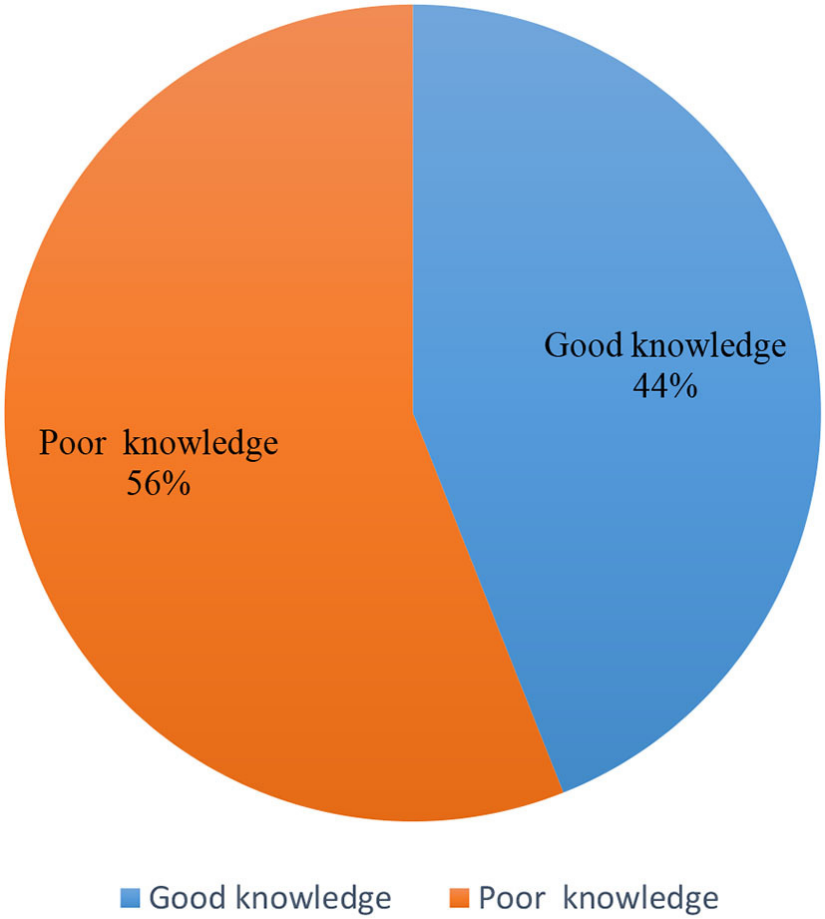
The levels of knowledge of thyroid.

Table 3 describes the participant's responses regarding the knowledge of warning signs for thyroid. In this study, slightly more than half of about 55.9% of participants believed that an unexplained lump or swelling might be a sign of cancer. Two-thirds said unexplained weight loss could be a sign of cancer, while 40.9% were uncertain. As for the symptoms such as a persistent change in the bowel, a sore that does not heal, a change in the appearance of a mole, and a persistent difficulty in swallowing, the responses recorded were 57.7% did not know and 22.1% yes; 56.1% did not know and 28.6% yes; 50.0% did not know and 31.5 % yes; 44.1% did not know and 42.7% yes, respectively (Table 3). In this study, 43.9% of the participants were found to have good knowledge, while 56% reported poor knowledge of warning signs for the thyroid (Figure 2).

**Table 3:** Frequency of correct answers regarding the knowledge of warning signs for thyroid.

**Characteristics**	**Correct answer Frequency *(n)***	**Correct answer Percentage (%)**
Iodine is required for the synthesis of thyroid hormones	205	28.3
Thyroid function impacts the menstrual cycle	298	41.2
Unexplained lump or swelling could be a sign of cancer	405	55.9
Persistent unexplained pain could be a sign of cancer	260	35.9
Unexplained bleeding could be a sign of cancer	303	41.9
A persistent cough or hoarseness could be a sign of cancer	260	35.9
A persistent change in bowel or bladder habits could be a sign of cancer	160	22.1
Persistent difficulty swallowing could be a sign of cancer	309	42.7
A change in the appearance of a mole could be a sign of cancer	228	31.5
A sore that does not heal could be a sign of cancer	207	28.6
Unexplained weight loss could be a sign of cancer	283	39.1

### Perception regarding chances of developing thyroid

Table 4 describes the participant's perceptions about developing thyroid. In terms of the perception of developing cancer, the highest percentage of participants (81.3%) believed that smoking increases the risk. A total of 73.3% of participants expressed that exposure to cigarette smoke from others increases cancer risk and 35.4% of participants were neutral about the statement that eating less than five portions of fruit and vegetables a day increases the risk of cancer. In total, 39.5 % of the participants positively responded that < 30 min of moderate physical activity five times a week can lead to cancer. Over two-thirds of the participants opted for neutral when it came to infection with HPV (Human Papillomavirus). More than 40% of the participants were neutral regarding statements like being overweight (BMI over 25), getting sunburnt as a child twice, and being over 70 years old for the possibility of developing cancer (Table 4).

**Table 4:** Perception regarding chances of developing thyroid.

**Variables**	**Strongly agree *n* (%)**	**Agree *n* (%)**	**Neutral *n* (%)**	**Strongly disagree *n* (%)**	**Disagree *n* (%)**	**Mean ±(SD)**
Smoking any cigarettes at all	436(60.2)	153(21.1)	100(13.8)	14(1.9)	21(2.9)	2.81 ± (1.23)
Exposure to another person's smoking	276(38.1)	255(35.2)	141(19.5)	17(2.3)	35(4.8)	3.32 ± (1.35)
Eating less than five portions of fruits and vegetables a day	85(11.7)	143(19.8)	256(35.4)	94(13.0)	146(20.2)	3.42 ± (1.57)
Eating red or proceed meat and junk food	111(15.3)	195(26.9)	264(36.5)	68(9.4)	86(11.9)	3.58 ± (1.65)
Being overweight	103(14.2)	164(22.7)	330(45.6)	49(6.8)	78(10.8)	3.51 ± (1.52)
Getting sunburnt	92(12.7)	142(19.6)	318(43.9)	79(10.9)	93(12.8)	3.37 ± (1.52)
Being over 70-year old	98(13.5)	165(22.8)	301(41.6)	57(7.9)	103(14.2)	3.53 ± (1.53)
Having a close relative with cancer	132(18.2)	169(23.3)	284(39.2)	58(8.0)	81(11.2)	3.23 ± (1.22)
Infection with HPV (Human Papillomavirus)	87(12.0)	81(11.2)	462(63.8)	39(5.4)	55(7.6)	3.07 ± (0.92)
Doing < 30 min of moderate physical activity five times a week	139(19.2)	147(20.3)	240(33.1)	86(11.9)	112(15.5)	3.13 ± (1.27)

### Differences between knowledge and perception scores and participant characteristics

Table 5 describes the differences between knowledge and perception scores and participant characteristics. According to findings, more than one-third of the Saudis about 43.9% (*n* = 318) have good knowledge, while 405(55.9%) of them were found to have poor knowledge. The mean knowledge score of the thyroid was found to be 4.1(SD=3.09), (Range 0–11) while the score of the mean perception was 33.02(SD = 6.41) (Range 0–41). Previous knowledge of thyroid disease has significantly affected the mean knowledge scores of Saudi adults (*t* = 5.08; *p* = 0.0001). The gender of the participant and the presence of chronic diseases were found to have no impact on the knowledge score of the thyroid disease (*t* = −1.18; *p* = 0.235; *t* = 1.005; *p* = 0.315) as shown in Table 5. Additionally, the perceptions score was not significantly different concerning the demographics of the participants (*p* = 0.05). Similarly, the education of participants is not significantly different concerning the knowledge or perception scores of the thyroid, while employment status is different with knowledge of the thyroid as shown in Table 2.

**Table 5:** Differences between knowledge and perception scores and participant characteristics.

	**Knowledge score**				**Perception score**			
**Participants characteristics**	**Mean (SD)**	***F* value**	*t*-value^*^	***p*-value**	**Mean (SD)**	*F* value^**^	***t*-value**	***p*-value**
**Gender**								
Male Female	3.85(3.28) 4.18(3.03)		−1.18	0.235^*^	32.6(6.68) 33.1(6.33)		−889	0.374^*^
**Age (full years)**								
18–22 23–25 26–30 31–34 35–40 45 and more	3.91(3.03) 4.52(3.26) 4.02(3.04) 4.46(2.97) 4.69(2.95) 5.09(3.71)	1.71		0.130^**^	32.8(6.28) 32.7(6.33) 32.2(7.10) 32.8(5.60) 35.9(6.78) 33.3(7.10)	2.05		0.069^**^
**Presence of chronic disease?**								
Yes No	4.44(3.23) 4.06(3.07)		1.005	0.315^*^	32.6(6.68) 33.0(6.38)		−0.562	0.574^*^
**Did you hear about thyroid disease?**								
Yes No	4.41(3.08) 3.01(2.88)		5.08^*^	0.0001	32.9(6.64) 33.1(5.54)		−0.303	0.7628^*^
**Place of residence**								
Central East West North South	4.13(3.12) 4.61(3.18) 4.05(3.11) 4.62(3.30) 3.90(2.95)	0.51		0.729^**^	32.7(6.37) 31.0(6.89) 33.5(5.97) 34.2(7.32) 32.8(6.78)	1.21		0.304^**^
**Professional class**								
Working Self-employed Housewife Students Unemployed	3.77(2.9) 4.78(3.1) 3.56(2.9) 4.08(3.6) 4.71(3.1)	5.41		0.0001	32.6(5.8) 33.5(7.0) 33.2(6.3) 32.0(6.5) 32.8(6.5)	0.788		0.533
**Schooling**								
Primary or below Secondary University	4.08(3.03) 4.18(3.30) 4.08(3.04)	0.056		0.946	32.5(6.13) 32.8(6.25) 33.5(6.69)	1.691		0.185

* t-test;

** ANOVA.

## Discussion

The incidence of metabolic disease has been increasing in recent years, which has made individuals pay more attention (16, 17). In particular, the prevalence of obesity, thyroid, and diabetes are on the rise in both Saudi Arabia and other international countries (16–19), which are significantly associated with multiple comorbidities, which in turn raise the mortality and disability rates over the world. There is not much literature available nationally and internationally about the knowledge and perceptions of individuals toward thyroid disease; however, most of the literature reported was identified in other regions of Saudi Arabia and neighboring countries (1, 5, 10). This study adds a significant contribution to identifying factors, which could contribute to the prevalence of thyroid among the young population in Saudi Arabia. It will serve as a baseline study for further studies looking at developing targeted interventions and services to reduce the risk of predisposing factors or help in the management of diseases to lead a healthy lifestyle.

In this study, 43.9% of the individuals reported having good knowledge of the thyroid. A similar study by Almuzaini et al. reported that 57.32% of Saudi adults reported having good knowledge of the thyroid (20). On the contrary, another recent study reported moderate knowledge of the thyroid (56%) (20). Although Alqahtani in 2021 reported moderate knowledge of 41.5% of the thyroid (5), Alyahya et al. (1) conducted a similar study in 2021 to assess the general knowledge about thyroid disease among the general population of eastern provenience of the same country and reported that 14.2% of the Saudis were knowledgeable about the disease. Therefore, poor knowledge of the thyroid is more common and is reported in many studies internationally (1, 10, 21, 22). This might be because the thyroid is most prevalent and typically has no apparent symptoms. If this condition is left untreated, severe complications may occur, in both males and females (23, 24).

The most common early signs of thyroid in this study were a lump in the neck or swelling (43.1%), followed by pain in the neck (24.6%), and difficulty in swallowing (23.8%). Similar results were reported by Almousa and Alotaibi on symptoms of thyroid disease as a neck swelling, constipation, and diarrhea (10). Another study by Alyahya et al. (1) identified fatigue (81.7%), neck swelling (70.6%), followed by weight gain (68.9%), and constipation and diarrhea (28.2%) (1). In comparison, the study by Rai et al. (22) reported weight gain or weight loss fatigue (61%). This variation in the findings might be due to the study design, questionnaire, living habits of the individuals, race, and ethnicity.

The most common justification among the participants in this study for the desirable candidate for thyroid was old age, followed by high radiation exposure, and family history, while the gender most affected by thyroid dysfunction was identified to be female (33.6%). These results were similar to a previously published study by Alqahtani in 2021, who reported that being female (58.4%), genetics (53.1%), and frequent exposure to radiation in childhood (37.5%) contributed to the thyroid (5). Similarly, another recent study reported that being female gender and a smoker is an important risk factor for thyroid diseases (1). Additionally, an earlier study reported that the antiarrhythmic medication, amiodarone, is another significant risk factor to induce thyroid dysfunction among the elderly (25).

In this study, the majority of the individuals agreed that smoking cigarettes or exposure to other smoke is positively effects the thyroid dysfunction. Similar results were reported by previous studies around the world (1, 5, 10, 13, 26). In addition, there was evidence that smoking leads to a decrease in the levels of thyrotropin and higher levels of thyroid hormones, which in turn causes the development of Graves' disease and hyperthyroidism (27). These findings necessitate additional awareness and knowledge programs among individuals about the harmful outcomes of smoking suggesting that smoking cessation, on the other hand, may lower the incidence and adverse events not only in the thyroid but also in multiple cancers associated with smoking implying that the effects of smoking may be reversed in persons who quit (27). Thus, current findings suggested that in order to prevent the morbidity and mortality linked to thyroid cancer and to limit the prevalence of thyroid, it is obligatory to create awareness among the public and should focus on educating people and raising mass knowledge of the disease's earliest symptoms and risk factors.

This study has some limitations. The first limitation is that it was an online web-based study, some elderly people or people who are technologically illiterate or lack access to the internet might find it difficult to participate, and this limited the response rate. According to earlier studies, the majority of people who suffer from chronic diseases such as thyroid are often young adults, thus it is still a useful strategy. The second is the study's self-reported design may have made our results less trustworthy. However, one can presume that thyroid-related clinical condition was accurately captured because the survey was anonymous and entirely voluntary. Despite the previous potential limitations, this study had a large sample size and sufficient response rates (>60%), which may be one of its strengths.

## Conclusion

These findings indicate that Saudi adults in Saudi Arabia are lacking some aspects of sufficient knowledge of thyroid diseases and their manifestation. More importantly, the prevalence of thyroid is on the rise compared with both national and international studies, which potentially predict an increase in negative consequences in the everyday life. The present results could serve as a support for healthcare facilities and providers to improve their patient counseling to encourage adherence to prescribed medications and to support the various strategies to control the thyroid. Therefore, we advocate the implementation of educational programs that teach patients and individuals about how to avoid the complications and incidence of thyroid diseases.

## Data availability statement

The original contributions presented in the study are included in the article/supplementary material, further inquiries can be directed to the corresponding author.

## Ethics statement

The studies involving human participants were reviewed and approved by IRB Approval of Research Project No. E-21-6371. The patients/participants provided their written informed consent to participate in this study.

## Author contributions

RA and AA designed the work and wrote this paper. WS, SA, and MA-R extracted and analyzed the datasets. S, SA, and MA-R interpreted the results and helped to revise the manuscript. All authors read and approved the final manuscript.

## Funding

The author(s) disclosed receipt of the following financial support for the research, and/or publication of this article. This study was supported by the Research Supporting Project, King Saud University, Saudi Arabia, (RSP-2021/378).

## Conflict of interest

The authors declare that the research was conducted in the absence of any commercial or financial relationships that could be construed as a potential conflict of interest.

## Publisher's note

All claims expressed in this article are solely those of the authors and do not necessarily represent those of their affiliated organizations, or those of the publisher, the editors and the reviewers. Any product that may be evaluated in this article, or claim that may be made by its manufacturer, is not guaranteed or endorsed by the publisher.
